# A Collection of Transgenic Medaka Strains for Efficient Site-Directed Transgenesis Mediated by phiC31 Integrase

**DOI:** 10.1534/g3.118.200130

**Published:** 2018-05-30

**Authors:** Tokiro Ishikawa, Satoshi Ansai, Masato Kinoshita, Kazutoshi Mori

**Affiliations:** * Department of Biophysics, Graduate School of Science; † Division of Applied Biosciences, Graduate School of Agriculture, Kyoto University, Sakyo-ku, Kyoto 606-8502, Japan

**Keywords:** medaka, phiC31, FLP, *Ac/Ds*, transgenesis

## Abstract

Genetic analysis is facilitated by the efficient production of transgenic strains expressing a DNA of interest as a single copy at a designated chromosomal location. However, technical progress toward this goal in medaka fish (*Oryzias latipes*), a vertebrate model organism, has been slow. It is well known that phiC31 integrase enables efficient site-directed transgenesis by catalyzing the recombination of an attP DNA motif in a host genome with an attB motif in a targeting vector. This system was pioneered in medaka using the *Sleeping Beauty* transposon system, and the attP site was established at three chromosomal locations. However, this number appeared insufficient with regard to genetic linkage between the attP-landing site and a genetically modified locus of interest. Here, to establish a collection of transgenic strains of medaka, we introduced an attP motif into the medaka genome using the *Ac/Ds* maize transposon system and established 12 independent transgenic strains harboring a single copy of the attP motif in at least 11 of the 24 medaka chromosomes. We designed an attB-targeting vector that was integrated efficiently and precisely into the attP-landing site, and with which the DNA of interest was efficiently transmitted to germline cells. Extraneous sequences in the integrants derived from the bacterial backbone of the attB-targeting vector as well as a transgenic fluorescence marker present in the attP-landing site were removable through flippase-mediated recombination. Further, an advanced targeting vector with a heart-specific recombination marker served as a useful tool for easily screening phiC31 integrase-mediated recombinant G_0_ embryos, leading to the efficient establishment of transgenic strains. Thus, our resources advance genetic research in medaka.

Small fish such as medaka (*Oryzias latipes*) and zebrafish (*Danio rerio*) serve as valuable vertebrate models for genetic research (Wittbrodt *et al.* 2002; Lieschke and Currie 2007). These fish, which are evolutionarily closely related to mammalian species, employ similar cellular responses to physiological and environmental stress (*e.g.*, the unfolded protein response against endoplasmic reticulum stress (Ishikawa *et al.* 2011; Cinaroglu *et al.* 2011; Thakur *et al.* 2011; Melville *et al.* 2011; Ishikawa *et al.* 2013; 2017a; b)). As the failure of such adaptation often impairs health, each serves as a model for a human disease (Dooley and Zon 2000; Wittbrodt *et al.* 2002). The transparent bodies of these fish allow fluorescent observations of larvae and adult organs, which has facilitated the development of many fluorescent transgenic fish strains (Rembold *et al.* 2006). Moreover, the recent development of genome editing technologies using targetable nucleases allows researchers to investigate gene function in these organisms much more easily than before (Doyon *et al.* 2008; Meng *et al.* 2008; Bedell *et al.* 2012; Hwang *et al.* 2013; Ansai *et al.* 2013; 2014; Ansai and Kinoshita 2014). Thus, small fish models facilitate genetic analysis of physiological and pathological processes that reflect those of humans (Witten *et al.* 2017, Wangler *et al.* 2017).

To generate transgenic fish, linearized or circular DNA vectors containing the DNA of interest (DOI) are microinjected into one-cell-stage embryos. Although the integration rate of this conventional approach is low, its efficiency can be improved by coinjection of meganuclease protein or transposase mRNA (Thermes *et al.* 2002; Kawakami 2005; Grabher and Wittbrodt 2007). However, transgenes introduced using these methods are randomly integrated into the host´s genome (Iyengar *et al.* 1996; Kuhn and Geyer 2003), and their copy numbers vary (Iyengar *et al.* 1996; Thermes *et al.* 2002; Kawakami *et al.* 2004). Moreover, the expression levels and expression patterns of transgenes often differ among strains harboring the same vector, requiring the availability of at least two strains per transgene (Kurauchi *et al.* 2008; Roberts *et al.* 2014).

The phiC31 integrase overcomes these disadvantages (Groth *et al.* 2004). The enzyme, which was originally found to be encoded in the genome of bacteriophage phiC31, catalyzes site-directed and unidirectional recombination between the two 34-bp motifs termed attB (attachment site bacterium) and attP (attachment site phage) (Thorpe and Smith 1998; Groth *et al.* 2000). Thus, once an attP motif is stably introduced into a host chromosome as an attP-landing site, this enzyme allows subsequent integration of a single copy of an attB-containing targeting vector into the attP-landing site (Groth *et al.* 2004). Transgenesis mediated by phiC31 integrase has been used in studies of *Drosophila melanogaster* (Groth *et al.* 2004; Bateman 2006; Venken *et al.* 2006; Bischof *et al.* 2007). This technique has also been pioneered in zebrafish and medaka fish using the *tol2* transposon system and the *Sleeping Beauty* transposon system, respectively, which was a milestone in genetic research in fish (Mosimann *et al.* 2013; Kirchmaier *et al.* 2013; Roberts *et al.* 2014).

To conduct a comprehensive analysis using phiC31 integrase-mediated transgenesis, it is important to consider the effects of genetic linkage. This often prevents the establishment of strains with the desired genotype, particularly if a locus modified by transgenesis or genome editing is too close to an attP-landing site to segregate. Therefore, it is better to establish transgenic strains containing a single copy of an attP motif in different chromosomes to serve as an experimental genetic reservoir so that an appropriate strain can be chosen. The number of attP-landing-site strains available for medaka and zebrafish is limited, however, at only 3 and 4, respectively (Mosimann *et al.* 2013; Kirchmaier *et al.* 2013; Roberts *et al.* 2014).

Here, we utilized the maize *Activator* (*Ac*)/*Dissociation* (*Ds*) transposon to establish 12 independent medaka attP-landing-site strains, each harboring a single copy of the attP motif within 11 chromosomes and one unidentified locus among the 24 medaka chromosomes. We then found that phiC31 integrase-mediated integration into these loci occurred with high efficiency and that an integrated gene was transmitted into a germline. DNA sequences integrated together with the DOI are removable using flippase (FLP)-mediated recombination, if necessary. Further, G_0_ embryos which have received phiC31 integrase-mediated recombinant sequences are easily identified by the heart-specific expression of fluorescent marker proteins encoded by the advanced attB-targeting vector.

## Materials and Methods

### Fish and imaging

The medaka southern strain Cab served as wild-type. Fish were maintained in a recirculating system with a 14:10 h light:dark cycle at 27.5°. All experiments were performed in accordance with the guidelines and regulations established by the Animal Research Committee of Kyoto University (approval number: H2819). Imaging of EGFP, mCherry, tagCFP, and Venus fluorescence was performed using a fluorescence stereomicroscope (Leica M205FA; Wetzlar, Germany) equipped with a camera (Leica DFX310FX) and image-acquisition software (Leica las AF) as well as a GFP3 filter (470/40 nm excitation filter and a 525/50 nm barrier filter), a DsRed2 filter (545/30 nm excitation filter and 620/60 nm barrier filter), a CFP filter (436/20 nm excitation filter and 480/40 nm barrier filter), and a YFP filter (500/20 nm excitation filter and 535/30 nm barrier filter).

In the heat treatment experiment, eggs incubated at 37° for 1 h were transiently heat shocked by transferring them to culture medium maintained at 42° in a dish, which was incubated at 37° for subsequent 2 h. Their fluorescence images were observed after 24 h at 28°.

### Vector

**Figure 1:**
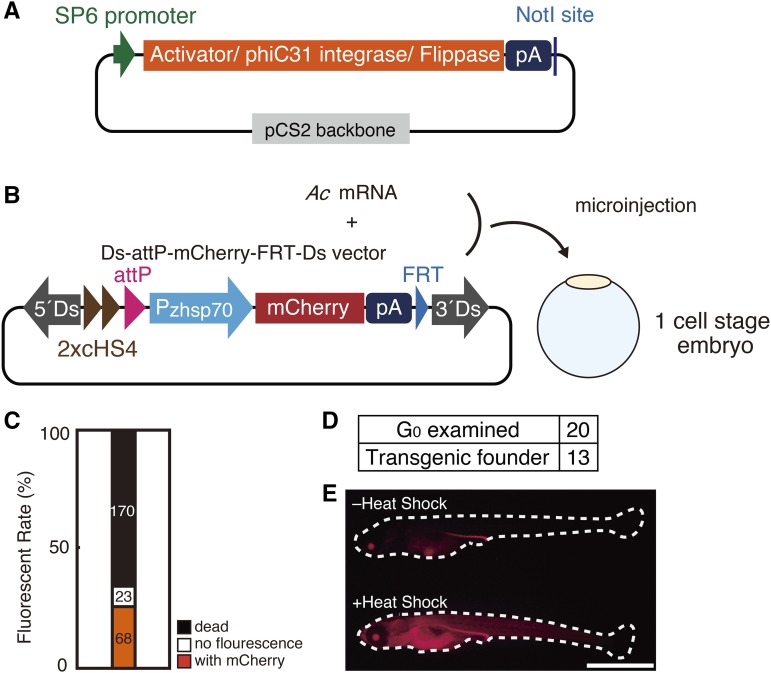
*Ac/Ds* transposon-mediated integration of an attP site into medaka chromosomes (A) Schematic representation of template vectors for generation of mRNA encoding *Activatior*, phiC31 integrase or Flippase. (B) Schematic representation of the Ds-attP-mCherry-FRT-Ds vector. Fertilized one-cell-stage embryos were microinjected with 10 ng/µl of this vector together with 25 ng/µl of *Ac* mRNA to establish attP-landing-site strains. (C) Ninety-one of 261 G_0_ embryos microinjected as in (B) survived and were subjected to heat shock at 5 dpf for observation of mCherry fluorescence. The numbers of dead, mCherry fluorescence-negative and -positive embryos are indicated in black, white, and orange columns, respectively. (D) Twenty fertile G_0_ fish were crossed with wild-type fish, and 13 were identified as transgenic founders that produced F_1_ transgenic offspring carrying the Pzhsp70-mChery-pA at the attP-landing site. (E) F_1_ transgenic fish were observed for mCherry fluorescence at 1 day after hatching with or without heat shock. The attP-landing site is in chromosome 13. Scale bar: 1mm.

The attP-landing vector (Figure 1B), Ds-attP-mCherry-FRT-Ds, comprises the 5′- and 3′-Ds ends amplified from pDsDELGT4 (Quach *et al.* 2015), a tandem repeat of chicken-derived insulator (2xcHS4) (Shimizu and Shimizu 2012), a 165-bp segment of the attP site (5′-GCTTCACGTTTTCCCAGGTCAGAAGCGGTTTTCGGGAGTAGTGCCCCAACTGGGGTAACCTTTGAGTTCTCTCAGTTGGGGGCGTAGGGTCGCCGACATGACACAAGGGGTTGTGACCGGGGTGGACACGTACGCGGGTGCTTACGACCGTCAGTCGCGCGAGCG-3′) from pBCPB+ (Addgene Plasmid 18940) (Groth *et al.* 2000), a 1.5-kb segment of the zebrafish hsp70 (zhsp70) promoter (Halloran *et al.* 2000) cloned from the genomic DNA of the zebrafish AB strain, the mCherry-coding sequence and an SV40 polyA signal from pmCherry-N1 (Clontech), a 48-bp segment of a synthetic FRT site (5′-GAAGTTCCTATTCCGAAGTTCCTATTCTCTAGAAAGTATAGGAACTTC-3′), and a minimal bacterial backbone derived from pPBIS19-mgfc:TagBFP-8xHSE:Cre (Okuyama *et al.* 2013).

The attB-targeting vector (Figure 3A) comprises a 274-bp segment of the attB site (5′-TCGACGATGTAGGTCACGGTCTCGAAGCCGCGGTGCGGGTGCCAGGGCGTGCCCTTGGGCTCCCCGGGCGCGTACTCCACCTCACCCATCTGGTCCATCATGATGAACGGGTCGAGGTGGCGGTAGTTGATCCCGGCGAACGCGCGGCGCACCGGGAAGCCCTCGCCCTCGAAACCGCTGGGCGCGGTGGTCACGGTGAGCACGGGACGTGCGACGGCGTCGGCGGGTGCGGATACGCGGGGCAGCGTCAGCGGGTTCTCGACGGTCACGGCGG-3′) amplified from pBCPB+ (Addgene Plasmid #18940) (Groth *et al.* 2000), the insulator sequence 2xcHS4, the FRT site, and a minimal bacterial genomic backbone.

The advanced attB-targeting vector with a heart-specific expression marker, (attB/Pzcmlc2-tagCFP, Figure 5A) contains a 0.9-kbp segment of the zebrafish cmlc2 promoter (Pzcmlc2) cloned from the genomic DNA of the zebrafish AB strain and a tagCFP-coding sequence with a BGH polyA signal derived from pTagCFP-mito (evrogen) between the attB site and the vector backbone of the attB-targeting vector.

The attB-Polubiquitin-Venus/Pzcmlc2-tagCFP vector (Figure 5C) contains a 3.1-kbp segment of the medaka fish ubiquitin (olubiquitin) promoter cloned from genomic DNA of the medaka Cab strain and a Venus-coding sequence with BGH polyA signal as the DOI in the advanced attB-targeting vector.

For *in vitro* transcription of *Ac* mRNA, the pCS2+Ac plasmid (Figure 1A) was generated by subcloning the *Ac* transposase gene together with the nuclear localization signal NLSK5E (Emelyanov *et al.* 2006) into the pCS2+MT vector (Turner and Weintraub 1994). For *in vitro* transcription of phiC31 integrase mRNA and FLP mRNA, the vectors pCS2+PhiC31o (Figure 1A) and pCS2+FLPo (Figure 1A) were generated by subcloning the PhiC31o- and FLPo-coding sequences from pPGKPhiC31obpA (Addgene Plasmid #13795) and pPGKFLPobpA (Addgene Plasmid #13793) (Raymond and Soriano 2007), respectively, into the pCS2+MT vector (the lower case letter o of PhiC31o and FLPo indicates codons optimized).

### RNA preparation and microinjection

*Ac*, phiC31 integrase, and FLP expression vectors (Figure 1A) were linearized using *Not*I, purified using phenol-chloroform extraction, and used as template to synthesize capped mRNAs using the Message mMachine SP6Kit (Life Technologies, Gaithersburg, MD) as described previously (Ishikawa *et al.* 2013). The RNAs were purified using an RNeasy MinElute (Qiagen) kit and microinjected into one-cell-stage embryos at the indicated concentration. Microinjection was performed as described previously (Ishikawa *et al.* 2011).

### Southern blot analysis

Southern blot analysis was performed according to published standard procedures (Ninagawa *et al.* 2014). A DIG-labeled probe for detection of mCherry was amplified by PCR using mCherry cDNA as template with the primers 5′-tgggtcgacATGGTGAGCAAGGGCGAGGAG-3′ and 5′-tggctcgagCTTGTACAGCTCGTCCATGCC-3′, and labeled using the PCR DIG Labeling Mix (Roche).

### Inverse PCR analysis of the flanking genomic region of each attP-landing site

Genomic DNA was extracted from an F_1_ or F_2_ adult fish of each attP-landing-site strain using a phenol-chloroform method. Each genomic DNA (2 µg) was digested with *Pst*I or *Bgl*II and then circularized using Ligation High Ver. 2 (Toyobo). The ligated DNA was used as the PCR template and amplified with the primers 3Ds-inverse-FW1 and 3Ds-inverse-RV1 (Table S1) and the DNA polymerase KOD FX Neo (Toyobo). The amplification reactions were performed as follows: 94° for 2 min, 35 cycles at 98° for 10 s each, 55° for 20 s, and 68° for 5 min. Subsequently, a 400-fold dilution of each PCR product was subjected to a second PCR reaction using the primers 3Ds-inverse-FW2 and 3Ds-inverse-RV2 (Table S1) as follows: 94° for 2 min, 30 cycles at 98° for 10 s each, 55° for 20 s, and 68° for 5 min. The amplicons were purified using gel extraction and individually subjected to direct sequencing using the primer 3Ds-inverse-FW2, performed by Eurofins Genomics (Tokyo, Japan). The genomic position of each fragment was determined using BLAT searches of the MEDAKA1/oryLat2 assembly provided by the UCSC genome browser (https://genome.ucsc.edu/) and the Hd-rR genome assembly (ver. 2.2.4) (http://utgenome.org/medakav2/). The annotation information from Ensembl Medaka release 91 (http://asia.ensembl.org/Oryzias_latipes/) was used to identify genes adjacent to each fragment.

To analyze the sequence signature of the Ds integration site, upstream and downstream primers were designed according to the flanking genomic sequences obtained for each attP-landing site (Table S1). The upstream genomic sequence was amplified from each attP-landing site using each upstream primer and the primer mCherry-529FW, and the downstream sequence was amplified using each downstream primer and the primer 5Ds-RV (Table S1). The upstream and downstream amplicons were directly sequenced using the primers 3Ds-FW and 5Ds-RV (Table S1), respectively.

### Data availability

All transgenic fish generated in this study are available upon request. All constructs described in this study will become available through the National Bio-Resource Project (NBRP) (https://shigen.nig.ac.jp/medaka/) or Addgene (https://www.addgene.org/). Supplemental material available at Figshare: https://doi.org/10.25387/g3.6317840.

## Results and Discussion

### Generation of a collection of transgenic strains harboring a single copy of an attP motif using the maize Ac/Ds transposase system

To stably introduce an attP motif into various chromosomes of medaka, we employed the maize transposon *Ac/Ds* system that functions in vertebrate species (Emelyanov *et al.* 2006; Froschauer *et al.* 2011; Boon Ng and Gong 2011). The *Ac* transposase recognizes the 5′- and 3′-Ds elements and transposes the DNA sequence between them in a vector into the host genome as a single copy at random positions.

We constructed the Ds-attP-mCherry-FRT-Ds vector containing, in 5′ to 3′ order, the 5′-Ds element, a tandemly repeated chicken insulator sequences termed hypersensitivity site 4 (2xcHS4 insulator) to prevent the effects of nearby enhancer activity, silencer activity, or both, an attP motif, the mCherry-coding sequence flanked by the promoter for zebrafish heat shock protein 70 and the SV40 polyA (pA) signal (Pzhsp70-mCherry-pA), a flippase recognition target (FRT) motif for later removal of extraneous sequences from the host genome, and the 3′-Ds element (Figure 1B). The mCherry served as a transgenic marker, which is constitutively expressed in the crystalline lens and becomes ubiquitously expressed upon heat shock (Blechinger *et al.* 2002) (see Figure 1E).

**Figure 2:**
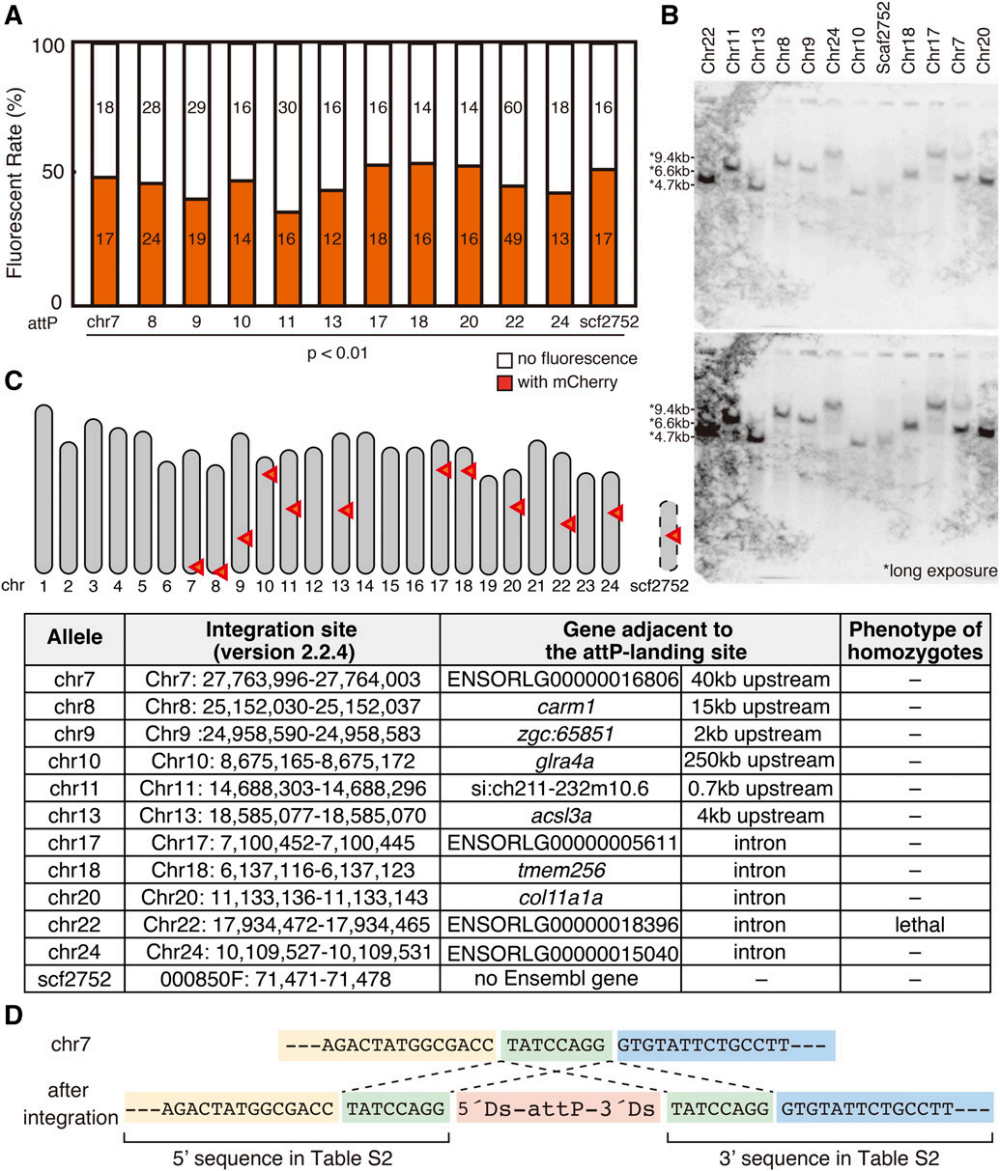
Establishment of a collection of transgenic fish harboring an attP-landing site (A) Each attP-landing-site strain was crossed with wild-type fish. The numbers of resulting embryos with or without constitutive mCherry fluorescence in lens are indicated as orange and white columns, respectively. *P* < 0.01 (binomial test) for rejection of the hypothesis that each genome harbored >1 transgene. (B) Genomic DNAs extracted from each attP-landing-site strain were digested with *Bgl*II and subjected to Southern blot analysis using a probe specific for mCherry. Molecular sizes indicated with asterisks were estimated in preliminary experments. (C) Schematic representation of the medaka chromosomes and the locations of the attP-landing sites indicated by red arrowheads. Detailed positions of each attP-landing site are shown below. (D) Example sequences of the attP-landing site in chromosome 7. The 8-bp sequence duplicated during transposition is shown in green.

We microinjected the Ds-attP-mCherry-FRT-Ds vector together with *Ac* mRNA into 261 one-cell-stage embryos to obtain 68 fish emitting ubiquitous mCherry fluorescence upon heat shock at 5 days post fertilization (5 dpf) (Figure 1C). The 68 fish were grown as generation 0 (G_0_) and most became fertile. After crossing with wild-type fish, 13 of 20 were identified as transgenic founders, which produced filial 1 (F_1_) fish emitting ubiquitous mCherry fluorescence upon heat shock (Figure 1D and 1E). After backcrossing several times with wild-type fish, we established 12 independent attP-landing-site strains, each of which harbors an attP motif as a single copy in the chromosomes (see Figure 2C).

Single-copy insertion of the Ds-attP-mCherry-FRT-Ds vector at a single locus in each strain was confirmed by the segregation ratio of the backcrosses (Figure 2A, approximately 50% of fish obtained by crossing each attP-landing-site strain with wild-type fish exhibited ubiquitous mCherry fluorescence upon heat shock) and Southern blot analysis (Figure 2B). We next conducted inverse PCR and subsequent sequencing of the products to identify each attP-landing site as described in Materials and Methods. The attP motif was integrated into various positions of 11 chromosomes and into a scaffold-attachment region that is not assigned to the medaka fish´s 24 chromosomes (Figure 2C, see Table S2 for actual upstream and downstream sequences of each attP-landing site). To eliminate the possibility that the insertion of the Ds-attP-mCherry-FRT-Ds vector might be deleterious to the functions of surrounding DNA regions, we incrossed heterozygotes of each strain and the resulting fish were genotyped after they reached maturity. Although we were unable to obtain homozygous transgenic fish harboring the attP-landing site in chromosome 22, homozygotes of other attP-landing-site strains were viable and fertile.

The *Ac/Ds* system belongs to the hobo-Ac-Tam3 (hAT) family of transposons, which duplicates an 8-bp sequence in the host genome and inserts a DNA sequence flanked by 5′-Ds and 3′-Ds between the duplicated sequences (Rubin *et al.* 2001), which becomes the attP-landing site. We confirmed that all attP-landing-site strains contained such sequences, suggesting that the *Ac* transposase mediated the integration of the Ds-attP-mCherry-FRT-Ds vector not via a random integration event (Figure 2D, Table S2). Twelve independent attP-landing-site strains were maintained as heterozygotes, homozygotes, or both.

### Efficient transgenesis using established attP-landing-site strains

**Figure 3:**
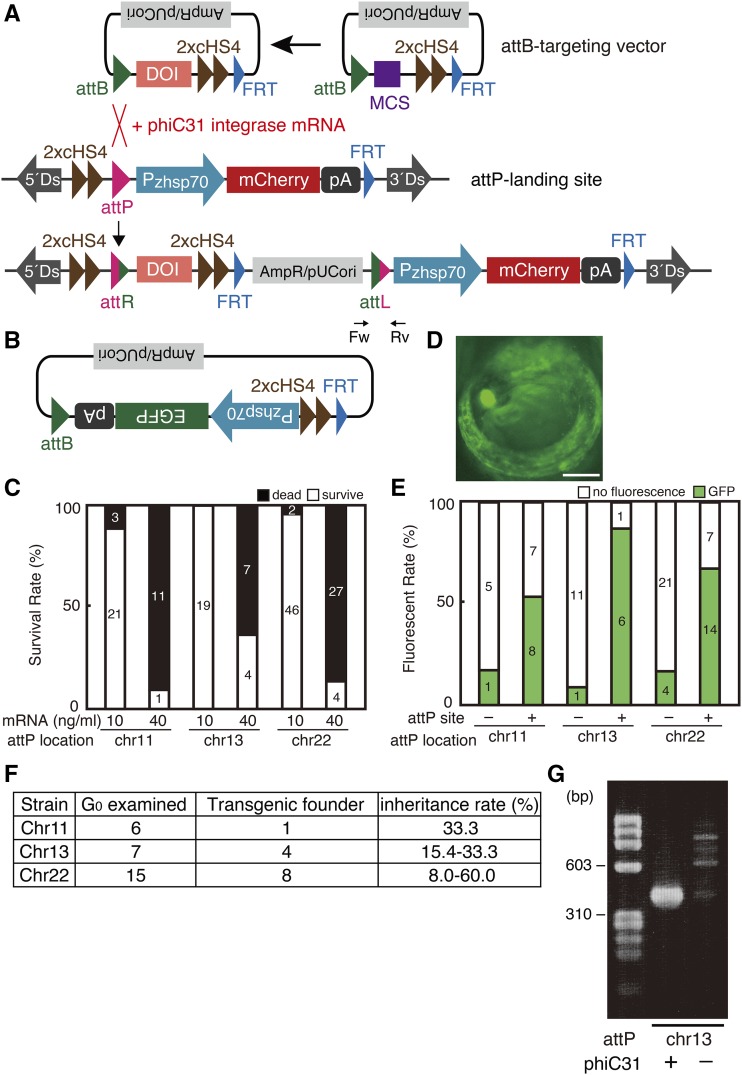
phiC31 integrase-mediated efficient transgenesis (A) Schematic representation of the attB-targeting vector and phiC31 integrase-mediated integration of this vector into attP-landing site. (B) Schematic representation of a targeting vector containing the Pzhsp70-EGFP-pA as the DOI. (C) Survival rates at 5 dpf of embryos microinjected with 10 ng/μl of the targeting vector shown in (B) together with 10 or 40 ng/μl of phiC31 integrase mRNA. (D) Typical EGFP fluorescence after heat shock of G_0_ embryos microinjected as in (C). Scale bar: 250 μm. (E) Detection rates at 5 dpf of EGFP fluorescence after heat shock of embryos that survived after microinjection with 10 ng/μl of the targeting vector shown in (B) together with 10 ng/µl of phiC31 integrase mRNA. (F) The number of transgenic founders in G_0_ fish as well as the inheritance rate of the GFP gene from G_0_ to F_1_. (G) Genomic PCR to detect the presence of attL in the attP-landing site using primers (Fw, Rv) shown in (A).

To perform phiC31 integrase-mediated transgenesis, we constructed the attB-targeting vector comprising, in 5′ to 3′ order, an attB motif, a multicloning site, the 2xcHS4 insulator, and an FRT motif. The DOI is to be inserted between the attB motif and 2xcHS4 insulator in this vector. Microinjection of this vector into one-cell-stage embryos together with phiC31 integrase mRNA inserts the DOI between the two 2xcHS4 insulators located upstream of the mCherry transgenic marker in the attP-landing site (Figure 3A).

To evaluate the efficiency of transgenesis, we inserted Pzhsp70-EGFP-pA into the attB-targeting vector as the DOI (Figure 3B) and microinjected the resulting plasmid (10 ng/µl) together with 10 ng/µl or 40 ng/µl of phiC31 integrase mRNA into one-cell-stage embryos obtained by crossing three attP-landing-site strains (*attP^chr11/+^*, *attP^chr13/+^*, and *attP^ch22/+^*) with wild-type fish. We found that 64–92% of embryos died within 5 days after microinjection with 40 ng/µl mRNA and that the mortality rates were reduced to 0–13% after microinjection with 10 ng/µl mRNA (Figure 3C). Therefore, we routinely microinjected an attB-targeting vector along with 10 ng/µl of phiC31 integrase mRNA in the experiments that follow.

Ubiquitous GFP expression was observed upon heat shock of microinjected G_0_ embryos harboring the attP-landing site in chromosome 11, 13 or 22 with much higher efficiencies (53–86%) than in microinjected wild-type embryos without the attP-landing site (8.3–16%) in all three (Figures 3D and 3E). The GFP gene integrated in G0 fish efficiently entered their germ lines, because 17%, 57% and 53% of the G0 from strains harboring the attP-landing-site in chromosomes 11, 13, and 22, respectively, were transgenic founders, which after crossing with wild-type fish produced F_1_ fish ubiquitously expressing GFP upon heat shock at 5 dpf (Figure 3F). The inheritance rates of the GFP gene from each transgenic founder to F_1_ fish were determined and are shown in Figure 3F.

Genomic PCR confirmed the presence of attL (Figure 3G) and attR (data not shown) in microinjected G_0_ embryos, which were produced only after attP/attB recombination (Figure 3A). Thus, phiC31-mediated recombination of the attB-targeting vector occurred specifically with high efficiency at the attP-landing site in microinjected G_0_ embryos.

### FLP-mediated locus cleanup

**Figure 4:**
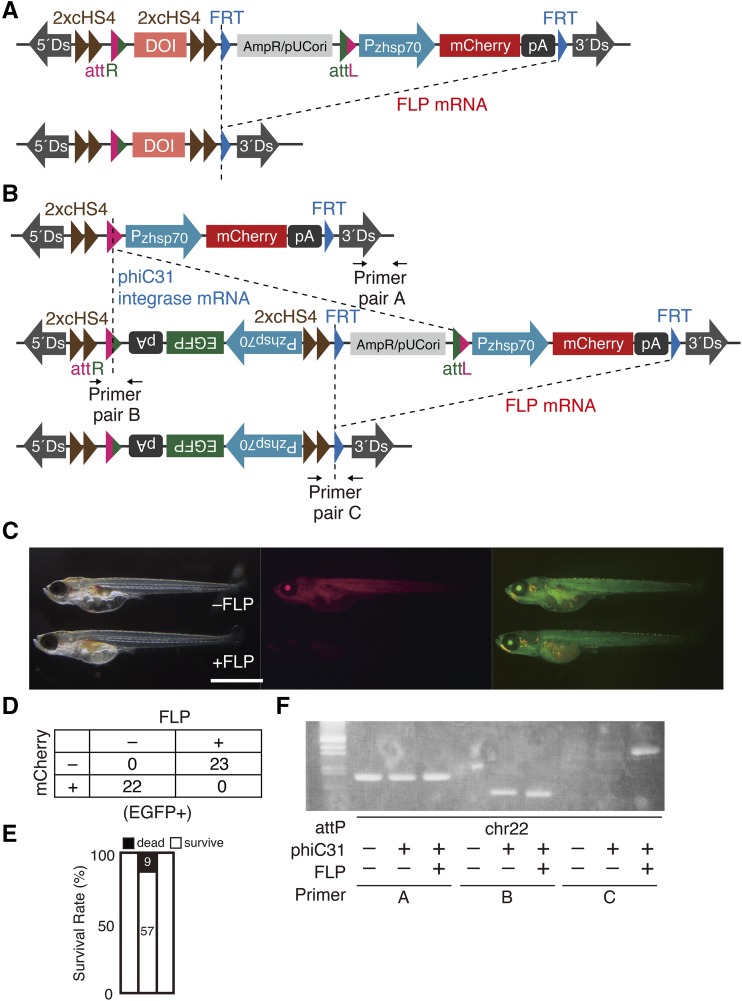
FLP-mediated removal of extraneous sequences (A) Schematic representation of FLP-mediated removal of extraneous sequences. (B) Schematic representation of the attP-landing site in chromosome 13 before and after phiC31 integrase-mediated integration of the Pzhsp70-mCherry-pA as well as subsequent FLP-mediated removal of extraneous sequences. (C) Typical observations of mCherry and EGFP fluorescence emitted by transgenic G_0_ fish carrying the Pzhsp70-mCherry-pA at an attP-landing site before (-) or after (+) FLP-mediated removal of extraneous sequences. Scale bar: 1 mm. (D) Transgenic fish harboring the Pzhsp70-mCherry-pA at the attP-landing site were incrossed, and one-cell-stage embryos were microinjected with 100 ng/µl of FLP mRNA. The 45 surviving embryos were observed at 5 dpf for mCherry and EGFP fluorescence after heat shock. (E) Survival rates at 5 dpf of embryos microinjected with 100 ng/μl of FLP mRNA. (F) Genomic PCR using the three primer pairs of three types of embryos with the attP-landing site structure shown in (B).

As described above, each attP-landing-site strain contains the transgenic marker Pzhsp70-mCherry-pA (Figure 3A) that emits fluorescence which may affect the observation of fluorescence emitted from the DOI. Further, after phiC31 integrase-mediated recombination, the fish integrants contained the ampicillin resistance gene and the pUC origin of replication derived from the attB-targeting vector (Figure 3A), which may affect the expression of the DOI (Tasic *et al.* 2011). Importantly, we designed the attB-targeting vector and the attP-landing site so that FLP allows the elimination of these extraneous sequences through recombination between the two FRT motifs (Figure 3A and 4A).

The attP-landing site in chromosome 13 in an F_1_ fish (Figure 3F) is shown in Figure 4B, and mCherry and EGFP were constitutively expressed in the lens and became ubiquitously expressed upon heat shock (Figure 4C and 4D, -FLP). To evaluate the efficiency of FLP-mediated removal, we microinjected FLP mRNA into one-cell-stage embryos obtained by incrossing this fish. We found that most embryos survived after microinjection of 100 ng/ul FLP mRNA (Figure 4E). We also found that mCherry fluorescence in all injected embryos became undetectable in the lens and whole body after heat shock, whereas EGFP fluorescence was not affected (Figure 4C and 4D, +FLP). When we performed genomic PCR analysis of the attP-landing-site strain in chromosome 22 using three pairs of primers (A, B and C) (Figure 4B), we found that the amplicon produced using primer pair C was detected only in embryos microinjected with FLP mRNA (Figure 4F). These data indicate that the mCherry marker and other extraneous sequences were correctly removed with extremely high efficiency through FLP-mediated recombination.

### Construction of an advanced attB-targeting vector harboring a recombination marker to enhance the efficiency of transgenesis

**Figure 5:**
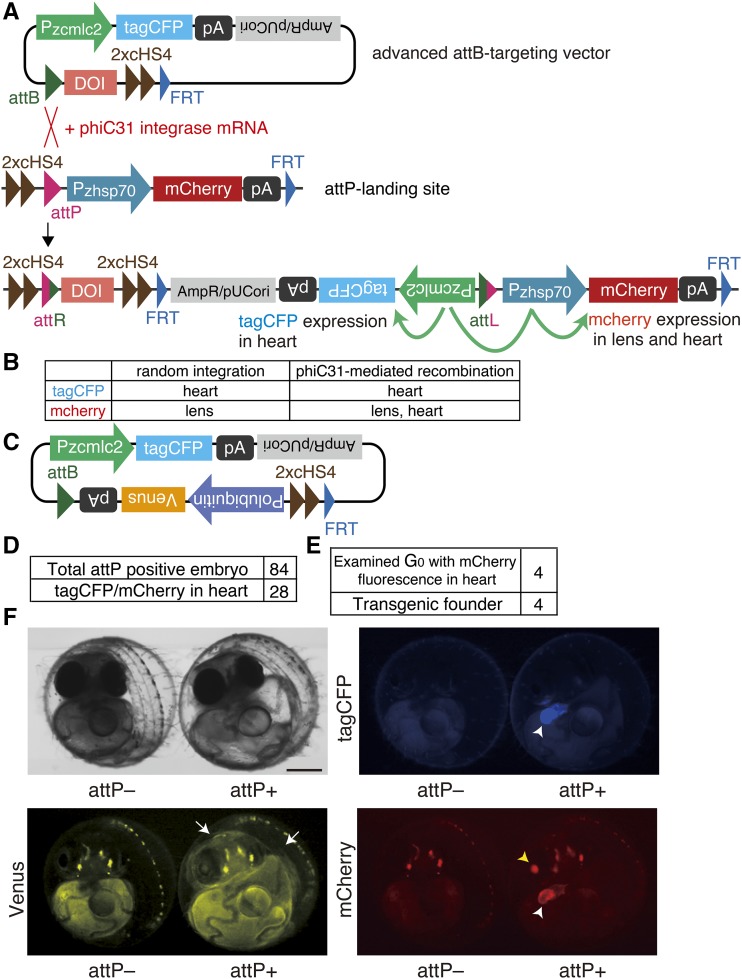
Advanced attB-targeting vector for easy detection of phiC31 integrase-mediated recombination (A) Schematic representation of the advanced attB-targeting vector and its integration into the attP-landing site mediated by phiC31 integrase. (B) Expected expression pattern of tagCFP and mCherry in transgenic G_0_ embryos produced by random integration or phiC31 integrase-mediated integration of the advanced attB-targeting vector. (C) Schematic representation of an advanced attB-targeting vector containing Polubiquitin-Venus-pA as the DOI. (D) The number of microinjected embryos at 5 dpf emitting mCherry fluorescence in lens (upper panel) as well as tagCFP and mCherry fluorescence in the heart (lower panel). (E) The number of transgenic founders emitting mCherry fluorescence in the heart in four G_0_ fish. (F) TagCFP, mCherry, and Venus fluorescence emitted by transgenic F_1_ progeny observed at 5 dpf. Left embryo: wild-type, Right embryo: transgenic fish. The white arrows indicate regions emitting Venus fluorescence. Note that the attP- embryo emitts autofluorescence. The white and yellow arrowheads indicate the heart and lens, respectively. Scale bar: 250 µm.

To further increase the system´s efficiency for generating transgenics, we constructed an advanced attB-targeting vector containing a tagCFP sequence flanked by the promoter of the gene encoding zebrafish cardiac myosin light chain 2 (zcmlc2) and SV40 pA (Pzcmlc2-tagCFP-pA) that was specifically expressed in the heart (Figure 5A). The Pzcmlc2-tagCFP-pA sequence is to be inserted into the upstream region of the Pzhsp70-mCherry-pA at attP-landing site in a head-to-head orientation through phiC31 integrase-mediated recombination (Figure 5A).

Interestingly, the zhsp70 promoter can be influenced by the activity of a nearby enhancer (Eichenlaub and Ettwiller 2011; ENCODE Project Consortium 2012). Therefore, if the advanced attB-targeting vector is correctly integrated into the attP-landing site via phiC31 integrase-mediated recombination, mCherry would be constitutively expressed in the lens and heart because of the enhancer activity of the zcmlc2 promoter. Thus, we can easily distinguish correctly targeted embryos from randomly integrated embryos by detecting the emission of tagCFP and mCherry fluorescence in the heart (Figure 5B). Moreover, the Pzcmlc2-tagCFP-pA sequence is removed together with the Pzhsp70-mCherry-pA sequence by FLP, if necessary, because they reside between the two FRT motifs after phiC31 integrase-mediated recombination.

To validate this system, we generated an advanced attB-targeting vector containing the Venus gene flanked by the medaka ubiquitin promoter and the SV40 pA (Polubiquitin-Venus-pA) as the DOI (Figure 5C). Male and female attP-landing-site strains were incrossed and we microinjected this vector together with phiC31 integrase mRNA in the embryos. Among 84 embryos with attP-landing-site (*i.e.*, emitting constitutive mCherry fluorescence in the lens) 28 embryos emitted tagCFP and mCherry fluorescence in the heart (Figure 5D). These fluorescent G_0_ embryos were raised and four were crossed with wild-type fish. Importantly, these four were transgenic founders, which produced F_1_ progeny that ubiquitously emitted weak Venus-yellow fluorescence, strong tagCFP-cyan fluorescence from the heart, and strong mCherry-red fluorescence from the lens and heart (Figure 5E and 5F). These data suggest that the zcmlc2 promoter did not affect the expression of Venus, the protein of interest, but did affect that of mCherry, which was originally under the control of the zebrafish hsp70 promoter.

In summary, we generated 12 independent attP-landing-site medaka strains using maize *Ac/Ds* transposon-mediated transgenesis, which allowed us to select an appropriate strain to avoid the effects of genetic linkage. Transgenic founders carrying a DOI can be produced quite efficiently through phiC31 integrase-mediated recombination between attP-landing site and attB present in standard and advanced targeting vectors. Extraneous sequences derived from the attP-landing site and the targeting vector are removed via FLP-mediated recombination, if necessary. Because the Ds elements in the attP-landing site remain intact, we can induce further *Ac*-mediated transposition of the introduced attP motif or integrated transgene to other genomic positions, if necessary. Using the protocols described here, this versatile transgenic system can be established in other species in which the *Ac/Ds* transposon system and phiC31 integrase function. Unlike the studies of Mosimann *et al.* 2013, Kirchmaier *et al.* 2013, and Roberts *et al.* 2014, our system does not utilize loxP or its variants, and we can therefore establish additional transgenic strains for the use of Cre/loxP recombination. Thus, a collection of the attP-landing-site strains and our vector system developed here show promise in serving as excellent genetic resources for the conduct of site-directed transgenesis in medaka fish.
